# From proton microdomains to protein migration: hypothesis for ΔpH-controlled PSII relocation

**DOI:** 10.1007/s11120-026-01240-2

**Published:** 2026-10-14

**Authors:** Matthias Ostermeier

**Affiliations:** Plant Molecular Biology, Faculty of Biology, Ludwig-Maximilians University Munich, Straße 2-4, 82152 Planegg-Martinsried, Germany

**Keywords:** ΔpH, Protonation, Thylakoid membrane, Thylakoid lumen, PSII

## Abstract

Thylakoid membranes are highly organized yet dynamic systems in which Photosystem II (PSII) complexes undergo continual assembly, repair and lateral redistribution. Here a hypothesis is proposed that local lumenal pH, generated by the trans-thylakoidal proton gradient and potentially exhibiting transient spatial heterogeneity, may contribute to the regulation of protein movement and PSII maturation within the thylakoid membrane. Five mechanistically distinct but potentially coupled routes by which ΔpH could influence protein dynamics are outlined: (i) pH-dependent modulation of lumenal protein-protein affinities; (ii) proton-sensitivity of lumenal proteases (e.g., CtpA) that gate processing and release of PSII subunits; (iii) proton-induced changes in membrane packing and stacking that alter diffusion pathways; (iv) formation of local proton microdomains that create chemically distinct niches; and (v) coupling of proton flux to electrostatic and lipid compositional shifts that bias protein localization. Together, these considerations suggest that ΔpH may act as a co-regulatory parameter integrating biochemical and biophysical cues in thylakoid organization and PSII homeostasis.

## Main

### In brief, function of thylakoid membranes and the role of Photosystem II

Thylakoid membranes are specialized internal membranes found both in cyanobacteria and in the chloroplasts of algae and plants. Their principal function is to host the light-driven reactions of oxygenic photosynthesis and to couple light energy capture to the formation of a transmembrane proton motive force (pmf). Structurally, cyanobacteria form an interconnected lamellar network, whereas in chloroplasts the thylakoid system is likewise continuous but differentiated into stacked regions (grana) interconnected by unstacked stroma lamellae. The membrane bilayer is enriched in galactolipids, including the non-bilayer-promoting monogalactosyldiacylglycerol (MGDG) and the bilayer-forming digalactosyldiacylglycerol (DGDG), as well as anionic lipids such as phosphatidylglycerol (PG), which collectively influence membrane packing and the activity and stability of embedded protein complexes (Demé et al. 2014; Fujii et al. 2025; Ostermeier et al. 2024).

The structural and dynamic behaviour of thylakoid membranes is often discussed in the context of the classic fluid-mosaic picture of biological membranes, but thylakoids require important refinements of that simple model (Singer and Nicolson 1972; Wilhelm et al. 2020). The Singer-Nicolson fluid-mosaic idea - proteins diffusing in a continuous lipid bilayer - captures the notion of lateral mobility, yet thylakoids are unusually protein-dense and organized into large, stable assemblies (for example PSII-LHCII supercomplexes), membrane stacks (grana) and microdomains that create lateral heterogeneity (Anderson 2012; Andersson and Anderson 1980; Engel et al. 2015; Fujii et al. 2025). As a result, the membrane is better described as a dynamic mosaic of semi-fluid domains: some components (mobile electron carriers, small peripheral subunits) retain relatively high freedom of movement, whereas larger complexes are sterically constrained, form transient or stable supercomplexes, or become confined by lipid phase behaviour, membrane curvature and protein-protein interactions (Brown 2012; Wilhelm et al. 2020). Functional processes - reversible phosphorylation, state transitions, assembly/disassembly during PSII repair and biogenesis, and changes in ionic or redox state - further modulate local packing and mobility, allowing the membrane to switch between more fluid and more ordered states as needed. This domain-centric view explains how thylakoids simultaneously support rapid energy transfer and long-lived spatial segregation and provides the physical language used in current work that links membrane composition and curvature to the localization, mobility and lifecycle of photosynthetic complexes.

Four large, multi-subunit protein complexes embedded in the thylakoid membrane carry out the primary energy-conversion chemistry: photosystem II (PSII), the cytochrome b_6_f complex, photosystem I (PSI), and the ATP synthase. In addition, mobile electron carriers (plastoquinone, plastocyanin) shuttle electrons between complexes and peripheral antenna systems gather sunlight and transfer excitation energy to the reaction centers (Komenda et al. 2024). The organization and antenna architecture differ between cyanobacteria and chloroplasts: cyanobacteria typically use phycobilisomes - large, water-soluble antenna assemblies - attached to the membrane, whereas chloroplasts of green algae and land plants use membrane-intrinsic light-harvesting complexes (LHCs) that participate in grana stacking and dynamic regulation of excitation energy (Neilson and Durnford 2010; Watanabe and Ikeuchi 2013).

PSII is the light-driven water: plastoquinone oxidoreductase and is central to oxygenic photosynthesis (Kern and Renger 2007). It is a dimeric, membrane-integral complex whose core contains the D1 and D2 proteins that bind the primary chlorophylls and the redox cofactors responsible for charge separation and electron transfer to plastoquinone. Two large chlorophyll-binding subunits (CP43 and CP47) form the inner antenna and scaffold additional pigments. On the lumenal side the oxygen-evolving complex (OEC) - a Mn₄CaO₅ cluster - catalyzes water oxidation to molecular oxygen and releases protons into the lumen; the OEC is stabilized by lumenal extrinsic proteins. Through sequential photooxidation of the Mn-cluster PSII extracts electrons from water, reduces plastoquinone to plastoquinol, and contributes protons to the lumenal compartment, thereby initiating the electron-transport chain and feeding into the proton gradient that drives ATP synthesis.

Physiologically PSII is both highly efficient and highly vulnerable: its reaction-center D1 protein is prone to photodamage under excess light, and cells maintain an active D1 repair and turnover cycle that involves selective proteolysis, replacement of damaged subunits, and reassembly of cofactor binding sites. In chloroplasts, PSII complexes are often concentrated in grana stacks where light capture and nonphotochemical quenching are regulated, whereas in cyanobacteria PSII distribution is determined by local membrane architecture and phycobilisome interactions (Krysiak et al. 2025; Rast et al. 2019). Together with PSI, cytochrome b6f and the ATP synthase, PSII forms a dynamic, spatially organized machinery that converts photon energy into chemical energy while balancing light harvesting, photoprotection and repair to maintain photosynthetic performance (Junge 2019).

### The heterogeneity of thylakoid membranes

**Fig. 1:**
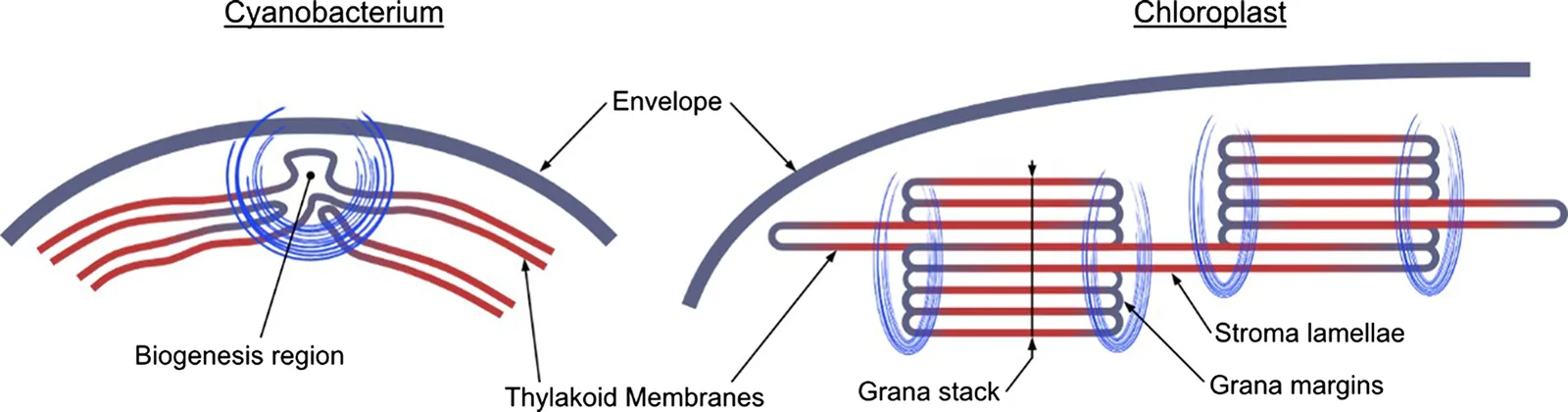
Thylakoid membranes in Cyanobacteria and Chloroplasts. Comparison of different thylakoid architecture. Red lines represent thylakoid membranes, blue highlights mark specialized regions characterized by low chlorophyll abundance and high biogenic activity.(adapted from Lübben et al. 2024).

The current picture is that PSII biogenesis does not occur uniformly across the thylakoid system but is spatially organized into specialized thylakoid subdomains where translation, co-translational membrane insertion and early assembly steps are concentrated (Ostermeier et al. 2025; Sakamoto 2025; Schottkowski et al. 2012). In cyanobacteria these subdomains have been identified as biogenesis regions or biogenic membranes (Fig. 1; thylakoid convergence regions in *Synechocystis sp*. PCC 6803) that are compositionally and functionally distinct from photosynthetically active membrane regions; they are enriched in assembly factors, ribosomes and early PSII assembly intermediates and often map to particular layers or margins of the thylakoid network rather than to the full lamellar surface (Ostermeier et al. 2025; Rast et al. 2019; Rengstl et al. 2011). In microalgae such as *Chlamydomonas reinhardtii*, a discrete translation zone (T-zone) adjacent to the pyrenoid serves as a primary locus for the local translation of chloroplast-encoded core proteins (notably D1), for chlorophyll synthesis and for the early steps of PSII assembly; the T-zone concentrates chloroplast mRNAs, ribosomes, pigment-biosynthetic enzymes and imported assembly factors so that nascent subunits and early intermediates accumulate there prior to redistribution (Schottkowski et al. 2012; Uniacke and Zerges 2007). In chloroplasts of multicellular algae and higher plants, analogous specializations have been localized primarily to stroma-exposed thylakoid regions and to grana margins/curvature zones (Fig. 1): many early assembly events and the translation/insertion of chloroplast-encoded core subunits take place on stroma lamellae or at grana margins before mature PSII complexes accumulate within grana stacks (Anderson 1989; Fujii et al. 2025; Krysiak et al. 2025; Puthiyaveetil et al. 2014; Sakamoto 2025).

All these subdomains have in common that they are nearly free of chlorophyll and, therefore, are less to non active in photosynthetic reactions (Fig. 1) (Lübben et al. 2024).

### Known ways how PSII navigates through the thylakoid membrane

How the nascent PSII complex relocates from the biogenesis site to its eventual predominant location (for example into grana stacks in higher-plant chloroplasts or into the more photosynthetically active membrane regions in cyanobacteria) is understood only in part and appears to combine predictable biophysical behavior of membrane proteins with regulated, factor-dependent steps. The experimentally supported sequence is broadly as follows: (1) early steps (translation, pigment insertion, formation of small subunit–core associations) occur at the biogenesis subdomain (Mullineaux and Mahbub 2023; Nickelsen and Rengstl 2013; Ostermeier et al. 2025; Rühle and Leister 2016); (2) late assembly reactions - including dimerization of PSII cores and association with extrinsic lumenal subunits and, in plants, with peripheral light-harvesting complexes - convert assembly intermediates into larger, more hydrophobic and sterically bulkier species (Albanese et al. 2017; Huang et al. 2021; Wu et al. 2025); (3) these maturation events change the diffusion properties and interaction partners of the complex and thereby its lateral distribution within the thylakoid membrane (Kirchhoff et al. 2007; Nath et al. 2013; Tomizioli et al. 2014). In other words, assembly-dependent changes in oligomeric state and in binding to antenna proteins are major characteristics of the complex’s redistribution. Consistent with a domain-centric picture, in vivo FRAP measurements in cyanobacteria report rapid lateral diffusion of phycobilisome antennae but an apparently immobile bulk PSII population under steady-state conditions (D ≈ 3 × 10⁻¹⁰ cm² s⁻¹ for phycobilisomes at 30 °C) (Sarcina et al. 2001). Interpretation of such FRAP results should account for the ensemble-and-timescale nature of the method (i.e. distinguish lateral diffusion from exchange, confined motion, immobile fractions and photophysical recovery) (Mullineaux 2004, 2008).

Superimposed on these passive, assembly-driven redistribution processes are active, regulated reorganizations of thylakoid architecture that facilitate directional movement. In chloroplasts, reversible phosphorylation of PSII core proteins (mediated principally by the STN8 kinase and reversed by specific phosphatases (Wunder et al. 2013) promotes PSII monomerization, loosening of grana stacking and migration of PSII out of tightly stacked grana into stroma-exposed lamellae where repair and late assembly steps can occur. The converse - dephosphorylation and re-association with LHCII - helps retention or reintegration of repaired/mature PSII back into grana (Theis and Schroda 2016). Thus phosphorylation/dephosphorylation cycles and the associated changes in membrane stacking and protein-protein interactions are among the best-documented mechanisms by which PSII relocates within the thylakoid network. Comparable regulation of lateral segregation has been observed in cyanobacteria, where membrane differentiation and specific protein-lipid environments correlate with spatial partitioning of assembly versus active photosynthetic sites (MacGregor-Chatwin et al. 2017).

Several molecular players assist relocation by stabilizing specific intermediate states or by promoting disassembly/reassembly steps that permit movement (Mabbitt et al. 2014). Examples include proteases and repair enzymes that remove damaged D1 and thereby generate assembly-competent monomers for reinsertion and reassembly, as well as chaperone-like assembly factors (Psb27, Psb28, Ycf48 etc.). These bind defined intermediates and are released only after a subsequent maturation step (Zabret et al. 2021), effectively escorting complexes through the spatial pathway from nascent insertion to functional residency. Structural and genetic studies have mapped many of these factors to the same subdomains where early assembly occurs, supporting a model in which the assembly factors both catalyze formation of intermediates and regulate when and where those intermediates become competent to diffuse into more densely packed, active membrane regions (Ostermeier et al. 2025; Rast et al. 2015; Rengstl et al. 2011; Stengel et al. 2012; Zabret et al. 2021).

Despite this overall picture, many mechanistic details remain unresolved and should be stated explicitly rather than inferred. It is not known whether PSII migration is driven by any dedicated directional transport machinery (i.e., a motor protein) or whether movement is entirely the consequence of changes in oligomeric state, binding partners and membrane physicochemistry combined with thermal diffusion (Theis and Schroda 2016). The precise physical route(s) taken by PSII (for example whether complexes move continuously through lateral diffusion, whether they hop between membrane microdomains, or whether they are transiently extracted into proteinaceous corridors) are incompletely characterized. The temporal coordination - how exactly the timing of pigment insertion, cofactor assembly, dimerization and LHC association is matched to the decision to remain at a biogenesis site or relocate - is still an active area of research, as is the detailed mapping of which specific lipids or curvature-sensing proteins define the biogenesis subdomains. Finally, while phosphorylation-dependent modulation of grana stacking and mobility is well supported, the full set of regulatory inputs that trigger relocation (light quality/intensity, assembly factor availability, redox signals, lipid composition changes) and how they are integrated spatially remains incompletely understood.

In sum, PSII biogenesis is spatially localized to specialized thylakoid subdomains where ribosomes, insertases and assembly factors concentrate early steps; maturation (dimerization, antenna attachment, cofactor loading) alters the complex’s properties and, together with regulated changes in membrane stacking (for example phosphorylation-mediated unstacking), drives redistribution toward the complexes’ principal functional sites. Many of the players and the broad sequence of events are now documented, but the field still lacks a complete, atomistic account of the physical mechanisms of directed movement and a full inventory of spatial cues that define biogenesis versus active zones - precisely these gaps current research is attempting to close. As outlined below, one possible player could be a heterogeneous proton distribution in the lumen of thylakoid membranes.

### A hypothetical new way of movement via the pH gradient

**Fig. 2:**
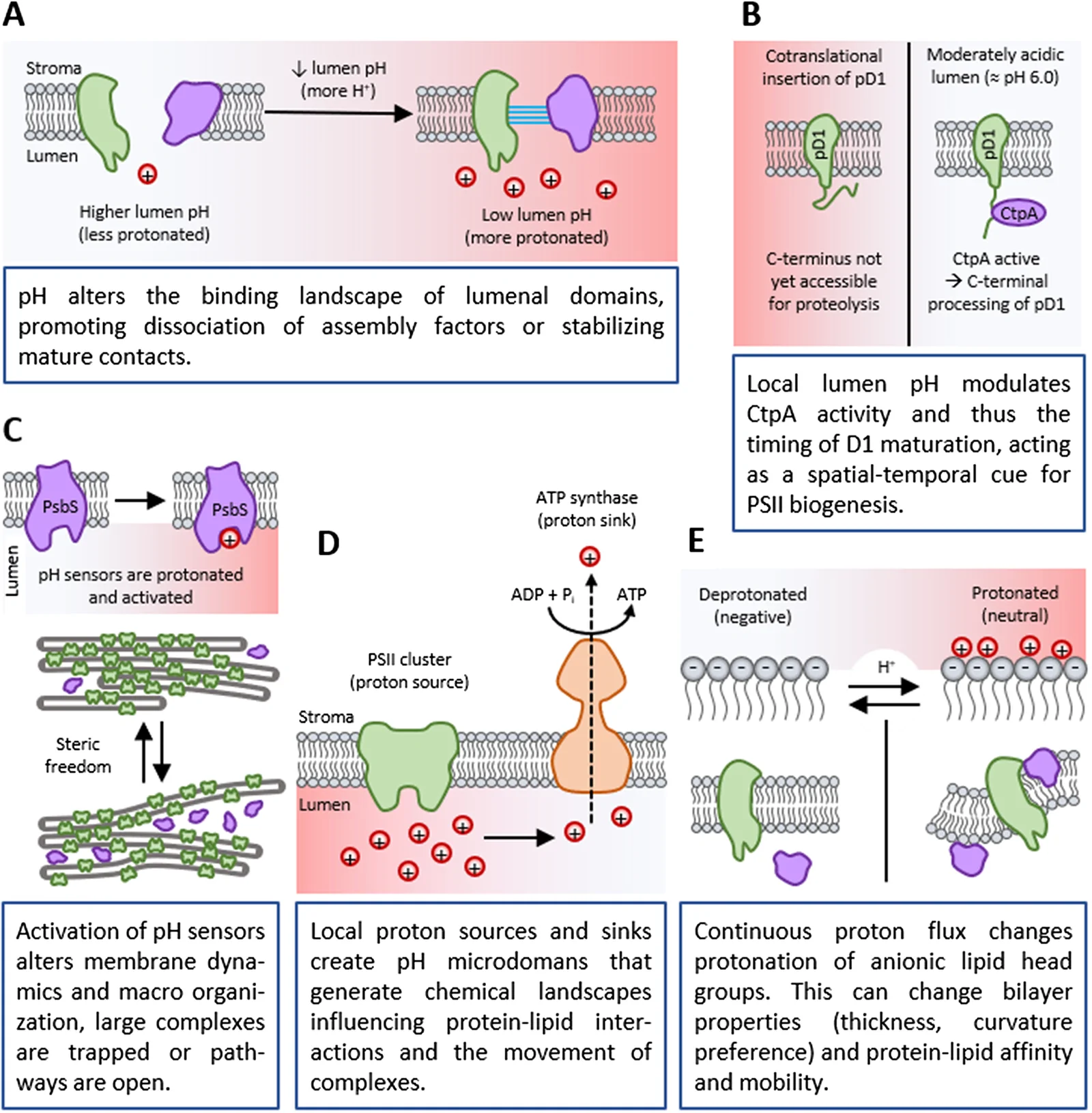
Hypothetical ways of membrane/protein interaction changes via transthylakoidal pH gradient (ΔpH). (**A**) Electrostatic switching of lumen-facing interactions. (**B**) Control of D1 maturation by pH-dependent proteolysis. (**C**) pH-driven reorganization of membrane packing and stacking. (**D**) Local proton sinks/sources and microdomain formation. (**E**) Coupling to ATP synthase activity and lipid/electrostatic environment

The light-driven water-splitting reaction of mature PSII releases protons into the thylakoid lumen and thereby contributes substantially to the trans-thylakoidal pH gradient (ΔpH) that, together with the membrane potential, comprises the proton-motive force used by ATP synthase (Lubitz et al. 2019; Tommos and Babcock 2000). Because nascent or immature PSII intermediates either lack a fully functional oxygen-evolving complex or perform water oxidation only weakly (Zabret et al. 2020), they will contribute far less to local lumen acidification than fully active centres - a difference that creates a physiological contrast between active PSII zones and sites of biogenesis/repair such as grana margins, T-zones, biogenic regions, and motivates the following hypotheses (Fig. 2). Historically, this idea sits within a long debate over whether thylakoid protons equilibrate rapidly with the bulk lumen or instead form localized, membrane-proximal domains. Early work by Theg and colleagues argued for proton subcompartmentation, while contemporaneous work explicitly framed the issue as localized versus delocalized proton behavior (Dilley et al. 1987; Hong and Junge 1983; Theg et al. 1982).

#### Electrostatic switching of lumen-facing interactions (affinity-switch hypothesis)

Protonation of lumen-exposed amino acid side chains at low lumen pH can alter the electrostatic surface of lumenal domains on PSII, assembly factors (e.g. Psb27 (Huang et al. 2021) and lumenal chaperones. Those pH-dependent changes in charge can alter affinities between components (for example by weakening or strengthening salt bridges or H-bonding networks) (Onufriev and Alexov 2013), thereby promoting dissociation of assembly factors or, conversely, stabilizing mature contacts. In this way, the local ΔpH could act as a permissive signal (Trinh and Masuda 2022) that alters the binding landscape as described by Zabret et al. (2021) (Fig. 2A).This may affect whether a partially assembled complex remains tethered at a biogenesis site or becomes free to diffuse into neighbouring membrane domains. Direct biochemical precedent for lumen-side pH sensing exists (for example PsbS and other lumen-facing pH sensors are protonated at low lumen pH and change conformation (Chiariello et al. 2023; Krishnan-Schmieden et al. 2021; Liguori et al. 2019)), so the general principle that protonation changes protein-protein interactions in the lumenal environment is well supported. 

Testing this idea will require correlating controlled lumen-pH changes with the binding dynamics of assembly factors and PSII intermediates in vivo.

#### Control of D1 maturation by pH-dependent proteolysis (timing-gate hypothesis)

The C-terminal processing protease CtpA, which removes the D1 precursor extension and thereby enables formation of the active Mn-cluster and downstream assembly steps, shows strong pH sensitivity in vitro and in membrane assays; different substrates or assay formats report distinct pH optima but experiments indicate increased activity on membrane-embedded pD1 at moderately acidic conditions (≈ pH 6.0) (Anbudurai et al. 1994; Chang et al. 2021) (Fig. 2B). This pH sensitivity is compatible with - and indeed complements - cotranslational insertion of pD1 (Ostermeier et al. 2025): cotranslational membrane insertion positions the pD1 C-terminus in the lumen but does not necessarily render that C-terminus immediately accessible or in the correct conformation for proteolysis (Zhang et al. 2000). Membrane context, substrate presentation and interactions with assembly factors can protect or mask the pD1 extension until other early assembly events are complete. The molecular basis of this pH dependence is not yet resolved and may involve effects on CtpA itself as well as on its membrane association and substrate presentation. The distinct pH profiles observed with synthetic and membrane-embedded pD1 support a contribution of membrane and substrate context rather than a simple intrinsic pH switch of the protease (Chang et al. 2021; Yamamoto et al. 2001). Because very low CtpA levels can still support pD1 processing in Synechocystis sp. PCC 6803, CtpA abundance alone should not be regarded as an established rate-limiting determinant of processing in vivo (Inagaki et al. 2001). The contribution of assembly factors such as LPA19 further indicates that CtpA-dependent processing can depend on the assembly context (Wei et al. 2010). Because biogenesis zones are expected to be less acidified than regions containing active, water-oxidizing PSII (Huokko et al. 2021; Järvi et al. 2013), a relatively alkaline local lumen would both reduce CtpA activity and favour retention of assembly-competent intermediates; conversely, lumen acidification (or changes in conformation/partner binding that expose the C-terminus) would accelerate CtpA-mediated maturation, permitting Mn-cluster formation, extrinsic subunit binding and dimerization and thereby promoting relocation of the complex. Thus, ΔpH can act indirectly as a spatial-temporal cue by modulating CtpA activity in concert with substrate accessibility and assembly cofactors; however, a direct in vivo demonstration that lumen pH alone times CtpA action remains to be conclusively shown 

Direct support for this model could come from experiments that simultaneously monitor lumen pH, CtpA activity, and the maturation state of D1 under changing physiological conditions.

#### pH-driven reorganization of membrane packing and stacking (mobility-release hypothesis)

Lumen acidification is a central trigger of photoprotective responses (e.g., qE) through protonation of pH sensor proteins such as PsbS and LHCSR (Ballottari et al. 2016; Nicol and Croce 2021; Niyogi et al. 2005); activation of these sensors and of associated pigment/antenna rearrangements is known to modify macro-organization of thylakoid membranes and to influence grana/stroma lamellae dynamics (Kirchhoff 2014b; Ünnep et al. 2020) (Fig. 2C). PsbS in particular controls the grana membrane organization (Betterle et al. 2009). Because lateral diffusion of large membrane complexes is strongly constrained by membrane packing, changes in stacking and local order induced by low lumen pH can increase or decrease the steric freedom of PSII assembly intermediates (Kirchhoff et al. 2008; Onoa et al. 2020) (Fig. 2C). Empirically, macromolecular crowding and packing constraints have been linked to the immobilization of large complexes such as PSII, while smaller or peripheral antennae remain mobile (Kirchhoff et al. 2008; Sarcina et al. 2001). For example, lumen acidification that promotes unstacking or local loosening could create pathways through which matured or repair-competent complexes more readily move into photosynthetically active domains, whereas conditions favouring tighter stacking could trap complexes (Kirchhoff 2014b; Li et al. 2020; Van Eerden et al. 2017). The established link between lumen pH, PsbS protonation and membrane reorganization supports this hypothesis, though direct observation that pH-induced reorganizations drive directional relocation of individual PSII assembly intermediates is still lacking. 

Future work combining ΔpH manipulations with high-resolution measurements of membrane architecture and PSII mobility should reveal whether such structural changes facilitate complex relocation.

#### Local proton sinks/sources and microdomain formation (microgradient hypothesis)

Local proton sinks and sources do not necessarily imply a uniform lumen-wide pH field at sub-micrometre scales (Vershubskii et al. 2017). Rather, they may generate transient, geometry-dependent proton microdomains whose extent depends on membrane architecture, buffering, and the balance between proton production and consumption (Fig. 2D). These considerations build on the longstanding concept of localized proton domains in thylakoids (Theg et al. 1982; Theg and Junge 1983). The intrinsic buffering capacity of the thylakoid lumen was experimentally estimated at approximately 0.8–1 mM per pH unit at pH 7.2 and 60 mOsm under the conditions examined by Junge et al. (1979). PSII water oxidation releases four protons per O₂ formed, but the resulting pH change depends on lumen volume, PSII abundance and activity, and the relevant time scale (Lubitz et al. 2019). Lumenal proteins may further influence local proton chemistry; PsbO, for example, contains lumen-exposed carboxylate groups with pH-dependent protonation properties and dynamic protein-water hydrogen-bond networks that are consistent with local proton binding (Bondar 2022; Gerland et al. 2020). Lumenal carbonic anhydrases may also influence proton chemistry in an organism-dependent manner, as shown for CAH3 at the C. reinhardtii PSII donor side (Shutova et al. 2008). 

Comparable lateral pH heterogeneity has been measured in mitochondrial cristae: in live cells, cristae-targeted pH sensors resolved a ~ 0.3 pH-unit difference between complex IV and F_0_F_1_ ATP synthase, and later work extended this to pH profiles at F_0_F_1_ ATP synthase, complex IV, and the matrix, demonstrating that local pmf is spatially heterogeneous within a single inner membrane system (Rieger et al. 2014, 2021). Proton release from active PSII clusters and local consumption by ATP synthase or buffering by lumenal proteins may contribute to transient pH heterogeneity (Armbruster et al. 2017; Farci and Schröder 2023; Theg et al. 1988). Such microgradients could create local “chemical landscapes” that favour particular protein–lipid interactions (for example protonation-dependent lipid headgroup behaviour) and hence locally modulate membrane fluidity or the affinity of assembly factors for membrane sites (Garab et al. 2017; McKinnon et al. 2020). If biogenesis zones maintain a different luminal pH microenvironment than active photosynthetic patches, this would add a physico-chemical basis for spatial segregation and for the selective release of matured complexes into regions with the appropriate pH (Chotewutmontri and Barkan 2024; Ostermeier et al. 2025; Rast et al. 2015). There is theoretical and experimental work pointing to limited lateral equilibration of protons in the thylakoid lumen and to lateral heterogeneity of proton potential, but direct in vivo mapping of such microgradients and their effect on protein trajectories remains an open experimental challenge (Junge and Polle 1986; Kaňa et al. 2023; Vershubskii et al. 2017). 

Resolving this question will require direct visualization of lumenal pH landscapes together with spatial tracking of PSII assembly intermediates at sub-micrometre resolution.

#### Coupling to ATP synthase activity and lipid/electrostatic environment (force-modulation hypothesis)

Proton flux through ATP synthase both consumes local protons and is associated with local electric fields (Sekiguchi et al. 2024); gradients in proton flux (for example where ATP synthase is enriched) may influence the local electrostatic screening of membrane surfaces and alter the packing or lateral mobility of charged complexes (Junge 1989; Schöttler et al. 2015). A related conceptual framework is provided by the recent “protet” or protonic charge transfer model, which proposes that protonic charge may be transiently stored in and transferred through protein networks during energy conversion (Kell 2024). In addition, continuous proton flux alters the local protonation state of lipid head groups (notably anionic lipids abundant in thylakoids; Fig. 2E) (Yoshihara et al. 2021), which can change bilayer thickness, curvature preference and protein–lipid affinity (Bolik et al. 2022; Kooijman et al. 2003). Through these effects, the steady-state balance between local proton release by PSII and proton consumption by ATP synthase could modulate the physical membrane landscape that determines how easily large complexes diffuse (Dekker and Boekema 2005; Kirchhoff 2014a; Onoa et al. 2020). This idea builds on the centrality of ΔpH to chloroplast energetics and on evidence that protein diffusion is sensitive to membrane supramolecular organization; again, direct mechanistic proof that differential proton fluxes drive directed protein migration is not yet available (Johnson and Wientjes 2020). 

A critical test would be to determine whether local perturbations of ATP synthase activity produce measurable changes in membrane organization that correlate with altered PSII distribution.

Each of the hypotheses above links physically plausible effects of proton concentration and flux to changes in binding, maturation or membrane organization that could alter mobility (Kaňa et al. 2023; Komenda et al. 2024). However, current research lacks direct, high-resolution in vivo demonstrations of causal chains that run from a measured, localized lumen pH change → a specific molecular change (for instance CtpA activation or a defined change in lipid protonation) → and then to a measurable, directed relocation of PSII intermediates (Bos et al. 2024; Kaňa et al. 2023; Komenda et al. 2024). It is also not known whether ΔpH acts mainly as a local biochemical timing cue (e.g., by modulating CtpA and other lumen-facing enzymes), mainly as a physical modulator of membrane order and diffusion, or as an integrated signal combining both effects - and the relative importance of these routes may differ substantially between cyanobacteria, microalgae (e.g. T-zones), organisms containing complex plastids and higher plants, given the profound differences in thylakoid architecture, membrane organization, spatial compartmentalization and associated protein complements across these groups (Komenda et al. 2024; Mehra et al. 2024; Ostermeier et al. 2024). Finally, quantitative data on lumenal pH microheterogeneity at the spatial and temporal scales relevant to single-complex movements are still sparse (Kaňa et al. 2023; Vershubskii et al. 2017).

Together, these hypotheses suggest that local ΔpH is not merely a consequence of photosynthetic electron transport but may act as a spatial regulator of PSII maturation and localization. While each proposed mechanism is individually plausible, future work will need to determine their relative contribution and physiological relevance.

## Conclusion

PSII biogenesis and movement are governed by the interplay between intrinsic changes in the complex during maturation and the dynamic physical structure of the thylakoid membrane. Early assembly occurs in distinct membrane regions enriched in assembly factors, where incomplete PSII forms are retained until they acquire their full complement of cofactors and subunits. As maturation progresses, the complexes become more stable and more compatible with the densely packed, energy-converting domains of the membrane.

The transthylakoid proton gradient (ΔpH) provides a unifying physical parameter that could link energy conversion to this spatial organization. Variations in lumenal pH influence the charge state of exposed protein surfaces, modulate the activity of lumenal enzymes such as CtpA, and affect the packing and curvature of the membrane through protonation of lipids and proteins. These pH-dependent effects are able to locally change the affinity of PSII assembly intermediates for their surrounding environment, thereby promoting their retention in biogenesis zones or their release toward active membrane areas.

Together, these processes describe a coherent picture in which PSII localization is not determined by a single molecular switch but by the combined action of maturation-dependent changes within the complex and pH-driven modulation of the surrounding membrane. This integrated physical and biochemical view provides a consistent hypothesis for how thylakoid membranes coordinate energy conversion with the continuous renewal and spatial redistribution of PSII.

Accordingly, ΔpH should be considered a potential co-regulatory parameter that acts alongside phosphorylation, redox control, proteolysis, and membrane remodelling rather than as a singular explanatory principle. The relationship between ΔpH and these regulatory processes, including phosphorylation-dependent PSII remodeling, remains unresolved. Taken together, these observations suggest that any pH-based contribution to PSII relocation should be treated as a testable extension of the older localized-proton-domain framework rather than as a settled mechanism. The broader question of proton-coupled membrane energetics remained active in later Theg work on cpTat transport and direct ΔpH/electric-field measurements, underscoring that proton-dependent membrane organization is still an experimentally open problem (Alder and Theg 2003; Theg and Tom 2011).

Mitochondrial evidence finally supports the broader premise that spatially restricted proton gradients can act as local regulatory parameters at the membrane level, rather than as a strictly bulk-equilibrated signal (Rieger et al. 2014, 2021).

Future experiments combining lumenal pH measurements with spatial analysis of PSII intermediates could directly test the proposed framework. Ratiometric pHluorin is a particularly relevant starting point because it has already been targeted to the thylakoid lumen, whereas probes such as SypHer3s and pHTomato could be explored as alternative genetically encoded sensors after appropriate targeting and calibration (Ermakova et al. 2018; Li and Tsien 2012; Yang et al. 2017). Combining pH measurements with super-resolution approaches such as STORM or MINFLUX may provide a route to relate local pH states to PSII or assembly-factor distributions, but such combinations would require suitable labeling and imaging strategies to be established (Kaňa et al. 2023; Scheiderer et al. 2025). Cryo-electron tomography provides native spatial information on thylakoid architecture in *C. reinhardtii* and *Synechocystis* sp. PCC 6803, including through subtomogram averaging, while cryo-EM has resolved defined PSII assembly intermediates (Engel et al. 2015; Rast et al. 2019; Zabret et al. 2021). Causal tests should combine perturbations of ΔpH or the overall pmf with time-resolved measurements of pH, CtpA activity and PSII distribution.

## Data Availability

No datasets were generated or analysed during the current study.
